# Arthroscopic transosseous anchorless versus suture anchor repair for rotator cuff tears: a meta-analysis

**DOI:** 10.1186/s12891-026-09557-8

**Published:** 2026-02-07

**Authors:** Yuyan Na, Xi Ren, Zongbo Wang, Yuting Shi, Jingjuan Wang, Litian Zhang, Yizhong Ren

**Affiliations:** 1 Department of Sports Medicine, The Second Affiliated Hospital of Inner Mongolia Medical University, No.59, Horqin South Road, Saihan District, Hohhot City, Inner Mongolia Autonomous Region China; 2 School of Nursing, Inner Mongolia Medical University, Hohhot, Inner Mongolia Autonomous Region China; 3 Department of Radiology, The Second Affiliated Hospital of Inner Mongolia Medical University, Hohhot, Inner Mongolia Autonomous Region China; 4 Department of Medical Oncology, Inner Mongolia Autonomous Region People’s Hospital, Hohhot, Inner Mongolia Autonomous Region China; 5 Department of Rehabilitation Medicine, The Second Affiliated Hospital of Inner Mongolia Medical University, Hohhot, Inner Mongolia Autonomous Region China; 6 Quality Management Department, The Second Affiliated Hospital of Inner Mongolia Medical University, No.59, Horqin South Road, Saihan District, Inner Mongolia Autonomous Region Hohhot City, China

**Keywords:** Rotator cuff tears, Transosseous anchorless repair, Anchor repair

## Abstract

**Background:**

Arthroscopic transosseous anchorless repair (TOA) has re-emerged as an alternative to suture anchor repair (AR) for rotator cuff tears, offering potential biomechanical advantages and cost savings. However, comparative efficacy remains debated. This meta-analysis evaluates clinical and structural outcomes of TOA versus AR techniques.

**Methods:**

Following Preferred Reporting Items for Systematic Reviews and Meta-Analyses (PRISMA) guidelines, six databases were systematically searched until May 2025. Comparative studies reporting functional scores (Constant, ASES), range of motion (ROM), visual analog scale (VAS) pain score, and retear rates were included. Risk of bias was assessed using Cochrane RoB 2 for randomized trials and MINORS for non-randomized studies. Meta-analyses used mean differences (MD) or risk ratios (RR) with 95% confidence intervals (CI).

**Results:**

Eight included studies demonstrated no statistically significant differences in most outcomes. Pooled analysis revealed no statistically significant differences in functional recovery (Constant score: MD = 1.54, 95%CI: -0.08 to 3.16, *P* = 0.06; ASES score: MD = 1.64, 95%CI: -0.62 to 3.90, *P* = 0.15) or postoperative pain (VAS: MD = − 0.05, 95%CI: −0.36 to 0.26, *P* = 0.76). Retear rates were comparable between TOA (13.2%) and AR (15.6%) groups (RR = 0.84, 95%CI: 0.49–1.46, *P* = 0.55). However, TOA showed superior improvement in abduction ROM (MD = 10.51, 95%CI: 5.29–15.72, *P* < 0.0001), while external rotation, internal rotation, and forward flexion showed no inter-group differences. Subgroup analyses confirmed equivalent outcomes when TOA was compared specifically to double-row or single-row anchor techniques.

**Conclusion:**

This meta-analysis demonstrates that TOA yields comparable clinical and structural outcomes to AR for rotator cuff tears. The only observed advantage of TOA was a statistically superior improvement in abduction range of motion. Therefore, TOA represents a viable alternative to anchor-based techniques in clinical practice.

**Supplementary Information:**

The online version contains supplementary material available at 10.1186/s12891-026-09557-8.

## Background

Rotator cuff tear is a common cause of shoulder pain and dysfunction, with prevalence rising significantly with age and exceeding 54% in individuals over 60 years [1]. Anatomically, degenerative or traumatic tears of rotator cuff disrupt shoulder biomechanics, leading to restricted motion, nocturnal pain, and muscle weakness [2]. Traditional open surgery utilized transosseous suture techniques for rotator cuff repair, but these were associated with significant trauma and prolonged recovery [3]. With advancements in arthroscopic techniques, anchor repairs-including single-row (SR), double-row (DR), and suture-bridge configurations-have become mainstream, enabling minimally invasive tendon-to-bone fixation [2, 4, 5]. However, concerns regarding anchor-related complications (e.g., anchor pullout, cyst formation, revision difficulty) and high costs have renewed interest in anchorless transosseous techniques [6–8].

Arthroscopic transosseous anchorless repair (TOA) involves creating bone tunnels to directly suture tendons without anchor implantation [9]. This approach combines the biomechanical advantages of traditional open transosseous fixation with the minimally invasive benefits of arthroscopy. Biomechanically, TOA provides a larger tendon-bone contact, reducing localized stress concentration caused by anchors [10, 11]. Biologically, bone tunnels may stimulate mesenchymal stem cell recruitment and growth factor release, thereby enhancing local vascularity and improving the healing microenvironment [12]. Additionally, TOA eliminates anchor-related complications and reduces instrument costs by approximately 35% [6–8].

Despite these advantages, the comparative efficacy of TOA versus anchor repair (AR) remains debated [13–15]. Several studies have reported no significant differences in shoulder function, pain, or retear rates between the two techniques [13–15]. Notably, the X-box or dual-tunnel design of TOA closely resembles the biomechanical structure of DR repair, both aiming to achieve broad footprint coverage and high-strength tendon-bone healing [16]. Nevertheless, existing studies are predominantly single-center, small-sample, or short-term, with inconsistent conclusions.

Therefore, this meta-analysis aims to compare TOA and AR techniques by synthesizing outcomes related to functional scores, range of motion (ROM), pain, and retear rates. Furthermore, this review will clarify whether the structural similarity between TOA and DR translates to comparable clinical outcomes, thereby providing evidence to support surgical decision-making.

## Methods

### Study registration

This systematic review was prospectively registered with the PROSPERO database (registration number: CRD420251059535) and conducted in accordance with the Preferred Reporting Items for Systematic Reviews and Meta-Analyses (PRISMA) guidelines [17].

### Search strategy

Six electronic databases (PubMed, Cochrane Library, Embase, Web of Science, CINAHL, and Scopus) were systematically searched from inception to May 10, 2025, using a combination of Medical Subject Headings (MeSH) and free-text terms: (“Rotator Cuff Tear” OR “Rotator Cuff Injury”) AND (“Arthroscopy” OR “Arthroscopic Repair”) AND (“Transosseous Suture” OR “Anchorless Repair” OR “Bone Tunnel”) AND (“Suture Anchor” OR “Single-Row” OR “Double-Row”). The study selection process was performed independently by two reviewers (Y.N. and X.R.). First, duplicate records were removed. Subsequently, titles and abstracts were screened against the inclusion and exclusion criteria. Potentially eligible studies then underwent full-text assessment. Any disagreement at any stage was resolved through discussion, or, if necessary, by consultation with a third senior reviewer (Y.R.). Additionally, manual searches of reference lists from included studies and relevant reviews were conducted to identify any additional eligible studies.

### Inclusion and exclusion criteria

Studies were selected based on the PICOS (Participants, Interventions, Comparisons, Outcomes, Study design) framework [18]: Inclusion criteria: 1. Participants: Symptomatic patients with MRI-confirmed rotator cuff tears undergoing primary arthroscopic repair. 2. Intervention (TOA group): Arthroscopic transosseous anchorless repair. 3. Comparator (AR group): Arthroscopic SR or DR suture anchor repair. 4. Outcomes: At least one of the following: functional scores (Constant score, ASES score), ROM, Visual Analog Scale (VAS) pain score, or retear rate assessed by magnetic resonance imaging (MRI) or ultrasound. 5. Study design: Comparative clinical studies (randomized controlled trials (RCTs), cohort studies). Exclusion criteria: 1. Studies on patients with prior ipsilateral shoulder surgery, concurrent glenohumeral instability. 2. Biomechanical studies, cadaveric studies, reviews, case reports, letters, conference abstracts, and studies with unavailable full-text.

### Quality assessment

The risk of bias and methodological quality of included studies were assessed independently by two reviewers (Z.W. and Y.S.). RCTs were evaluated using the Cochrane Risk of Bias Tool (RoB 2) [19]. Non-randomized studies were assessed with the Methodological Index for Non-Randomized Studies (MINORS) (12-item checklist) [20]. Scores: 0–6 (very low), 7–10 (low), 11–15 (moderate), ≥ 16 (high quality). Two investigators independently performed assessments, with disagreements resolved by a third reviewer (J.W.).

### Data extraction

Two reviewers (X.R. and Z.W.) independently extracted: Study characteristics: Author, year, country, design, surgical technique, sample size; Patient demographics: Age, sex; Surgical details: TOA (e.g., X-box, dual-tunnel) vs. AR (e.g., SR, DR, suture-bridge) methods; Outcomes: functional scores, ROM, pain, retear rates.

### Statistical analysis

Meta-analysis was conducted using RevMan 5.3 (The Cochrane Collaboration). For continuous outcomes (e.g., functional scores, ROM, VAS), the mean difference (MD) with 95% confidence interval (CI) was calculated. For dichotomous outcomes (retear rate), the risk ratio (RR) with 95% CI was computed. Heterogeneity was assessed using the I² statistic, with values below 50% indicating acceptable heterogeneity for fixed-effects models, while higher values prompted the use of random-effects models. Subgroup analyses compared TOA vs. SR and TOA vs. DR. All statistical tests were two-sided, and a *P* value < 0.05 was considered statistically significant.

## Results

### Study selection

**Fig. 1:**
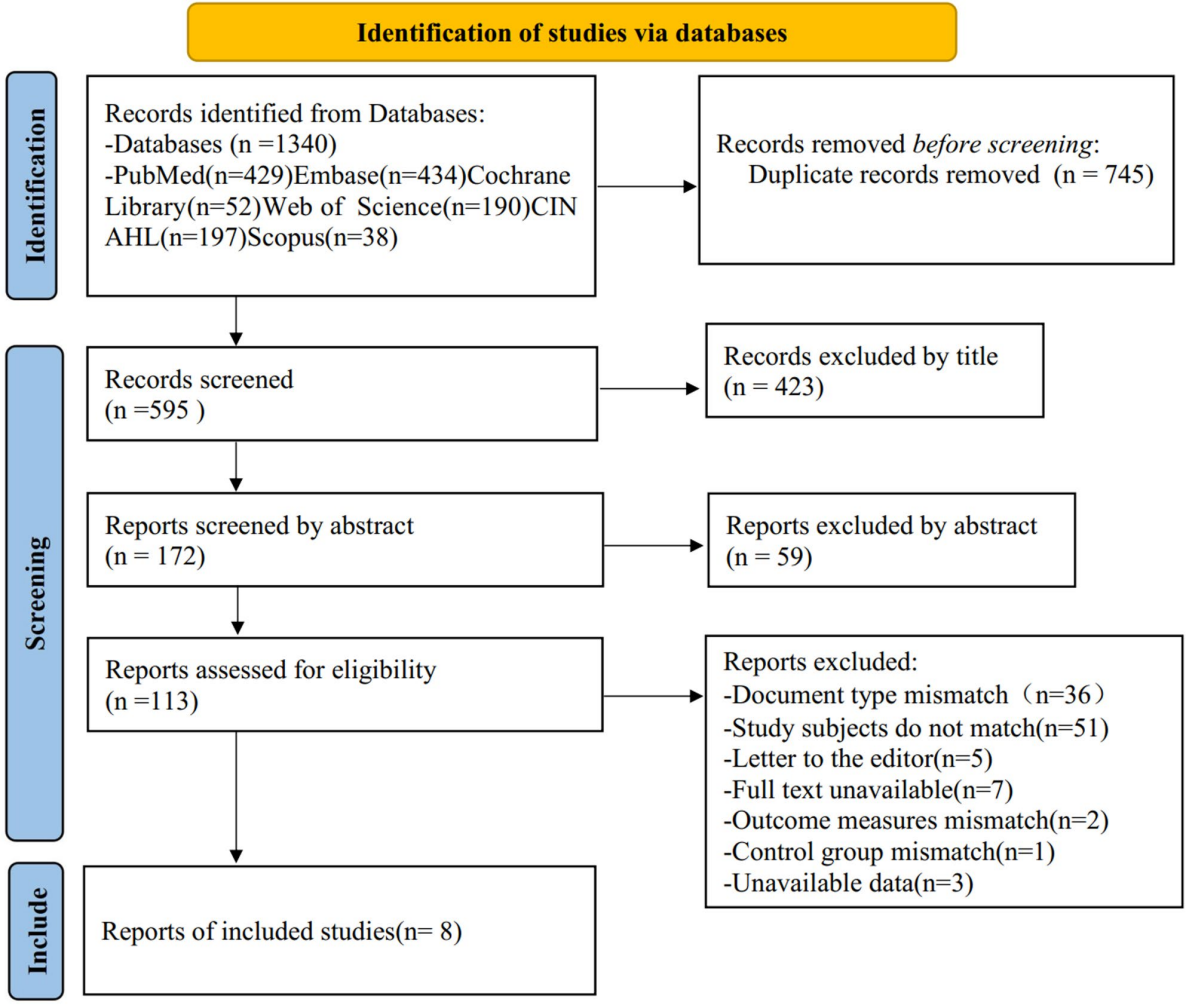
Flow diagram of study selection using PRISMA

The PRISMA flow diagram (Fig. 1) details the screening process. Initial database searches identified 1,340 records. After removing duplicates (*n* = 745) and irrelevant studies via title/abstract screening (*n* = 482), 113 full-text articles were assessed. Eight comparative studies met inclusion criteria [8, 13, 14, 21–25], comprising: 2 RCTs [14, 24] and 6 retrospective cohort studies [8, 13, 21–23, 25]. Total enrollment was 582 patients (TOA: 286; AR: 296). The main characteristics of the included studies are listed in Table 1.

**Table 1:** The main characteristics of all included articles

Study	Country	Year	Study design	Level of Evidence	Patients		Age		Gender (M/F)		Surgical type		Times		Outcomes
					C	I	C	I	C	I	C	I	C	I	
Binder et al. [21]	Austria	2022	Retrospective study	Ⅲ	25	45	61 ± 8	56 ± 9	21/4	22/23	DR	TOA	34 ± 6.8	29 ± 5.2	CS、VAS、Retear rate
Garofalo et al. [22]	Italy	2018	Retrospective study	Ⅱ	42	54	56.8 ± 8.7	58.7 ± 9.2	19/23	20/34	SR	TOA	24	24	CS、ASES、Retear rate
Hirakawa et al. [23]	Japan	2024	Retrospective matched control study	Ⅲ	42	42	68	67	20/22	20/22	DR	TOA	38	36	CS、ASES、VAS、ROM、Retear rate
Jeong et al. [8]	Korea	2023	Retrospective matched control study	Ⅲ	57	19	62.0 ± 10.3	62.3 ± 7.5	23/34	6/13	SR	TOA	36.9 ± 12.4	39.6 ± 10.6	ASES、VAS、ROM
Mesriga et al. [14]	Egypt	2025	RCTs	Ⅰ	31	31	56.4 ± 6.6	55.3 ± 5.4	10/21	17/14	SR	TOA	28.4 ± 3.8	29.1 ± 3.1	ASES、ROM
Randelli et al. [24]	Italy	2017	RCTs	Ⅰ	35	34	No report		No report		SR	TOA	15	15	CS、Retear rate
Sundar et al. [13]	India	2025	Retrospective matched control study	Ⅲ	20	22	60.7 ± 8.0	59.9 ± 7.5	13/7	13/9	DR	TOA	12	12	ASES、ROM
Fırat et al. [25]	Turkey	2020	Retrospective matched control study	Ⅲ	44	39	57.86 ± 7.81	57.03 ± 6.39	8/36	7/32	DR	TOA	32.95 ± 11.76	33.72 ± 12.14	VAS、CS

*RCTs* randomized controlled trials, *C* control group, *I* intervention group, *M* male, *F* female, *DR* double-row, *SR* single-row, *TOA* transosseous anchorless repair, *CS* Constant score, *ASES* American Shoulder and Elbow Surgeons, *VAS* visual analog scale, *ROM* Range of Motion

### Quality assessment

The overall methodological quality of the included studies was moderate to high. Quality assessment revealed one RCTs [24] had a high risk of bias in randomization, while other domains were low-risk (Table 2). Among non-randomized studies, the MINORS tool indicated high methodological quality for five studies (scores ≥ 16/24), with one study [21] rated as moderate quality (11/24) (Table 3).

**Table 2:** Risk of bias of included RCTs studies

Study	A1	A2	A3	A4	A5	A6	A7
Mesriga et al. [14]	Low	Low	Low	Low	Low	Low	Low
Randelli et al. [24]	High	Low	Low	Low	Low	Low	Low

A1: Random sequence generation; A2: Allocation concealment; A3: Blinding of participants and personnel;

A4: Blinding of outcome assessment; A5: Incomplete outcome data; A6: Selective reporting; A7: Other bias

**Table 3:** Risk of bias of included non-RCTs studies

Study	B1	B2	B3	B4	B5	B6	B7	B8	B9	B10	B11	B12	Total	Grade
Binder et al. [21]	2	1	1	2	0	2	1	0	2	0	2	2	15	Moderate
Garofalo et al. [22]	2	1	0	2	2	1	1	0	2	2	2	2	17	High
Hirakawa et al. [23]	2	1	1	1	1	1	1	2	2	2	2	2	18	High
Jeong et al. [8]	2	2	0	1	2	2	0	0	2	2	2	2	17	High
Sundar et al. [13]	2	2	1	2	1	2	0	0	2	2	2	2	18	High
Fırat et al. [25]	2	1	1	2	1	1	0	0	2	2	2	2	16	High

B1: A clearly stated aim;

B2: Inclusion of consecutive patients;

B3: Prospective collection of data;

B4: Endpoints appropriate to the aim of the study;

B5: Unbiased assessment of the study endpoint;

B6: Follow-up period appropriate to the aim of the study;

B7: Loss to follow up less than 5%;

B8: Prospective calculation of the study size;

B9: An adequate control group;

B10: Contemporary groups;

B11: Baseline equivalence of groups;

B12: Adequate statistical analyses

### Functional outcomes

**Fig. 2:**
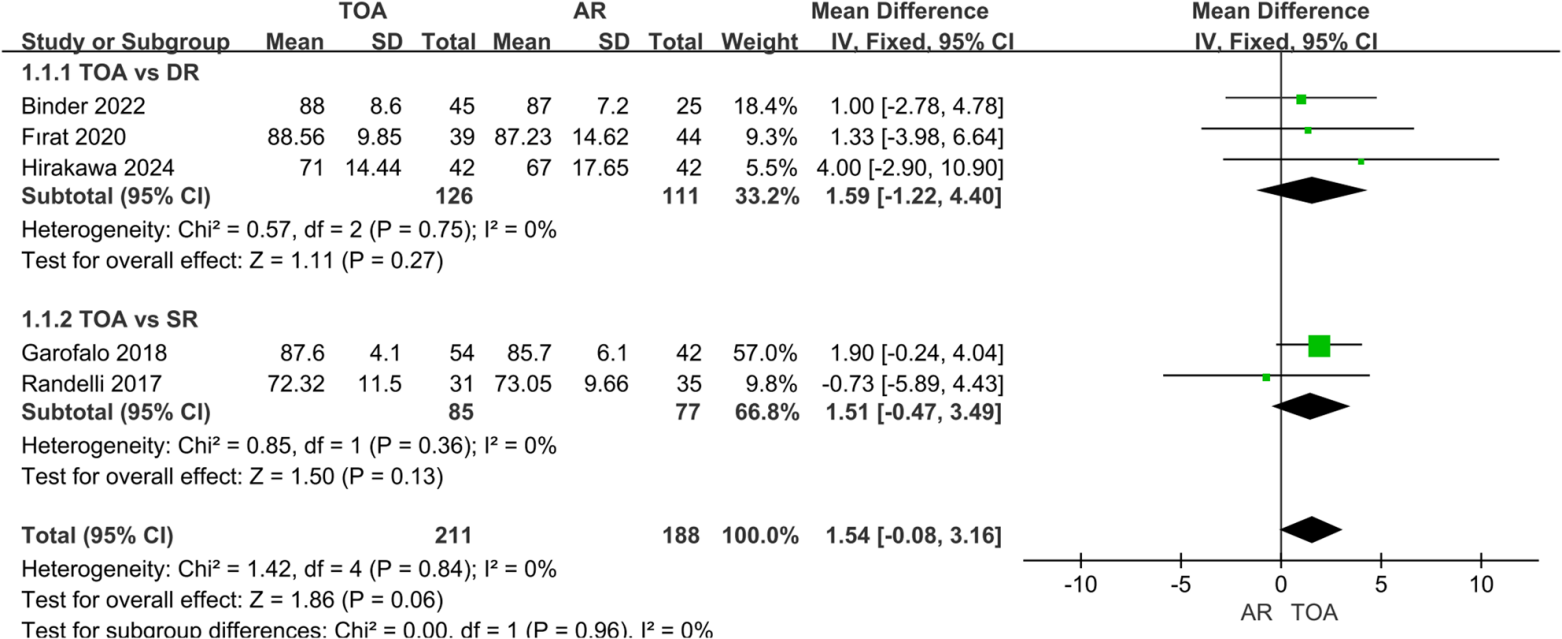
Forest plot comparing Constant scores between TOA and AR groups

**Fig. 3:**
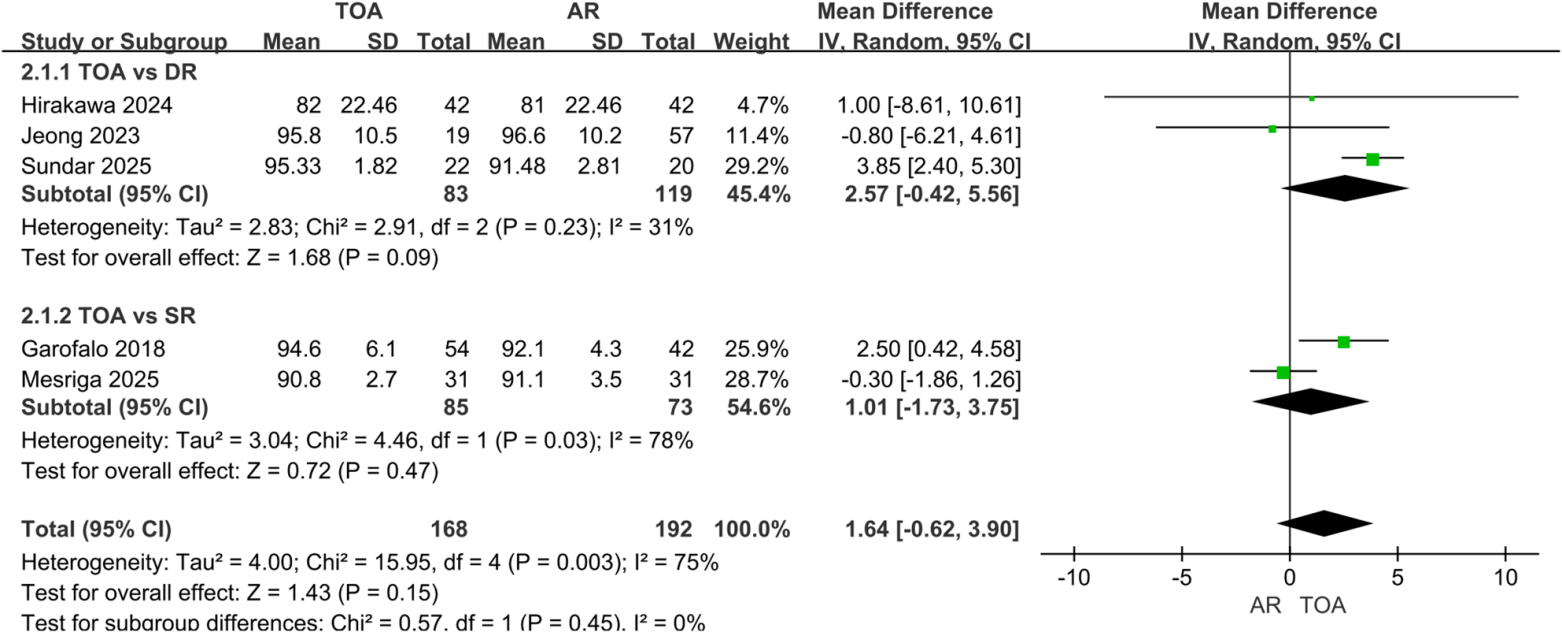
Forest plot comparing ASES scores between TOA and AR groups

Pooled analysis demonstrated no statistically significant differences between TOA and AR techniques in functional assessment. The Constant score (399 patients) showed a non-significant trend favoring TOA (MD = 1.54, 95% CI: -0.08 to 3.16, *P* = 0.06), with no heterogeneity (I² = 0%) (Fig. 2). Similarly, the ASES score (360 patients) revealed no significant group difference (MD = 1.64, 95% CI: -0.62 to 3.90, *P* = 0.15), though substantial heterogeneity existed (I² = 75%) (Fig. 3). The heterogeneity may reflect variations in study design, follow-up length, rehabilitation protocols, and surgical technique details across studies. Although a random-effects model was applied, these factors should be considered when interpreting the pooled estimate. Subgroup analyses comparing TOA specifically with DR or SR anchor configurations likewise showed no significant differences in either functional score.

### Range of motion

**Fig. 4:**
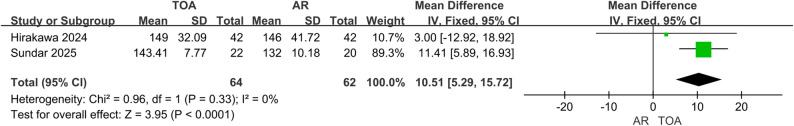
Forest plot comparing abduction range of motion between TOA and AR groups

Meta-analysis of ROM outcomes indicated comparable recovery between groups for most movements. External rotation (264 patients; MD = -0.12, 95% CI: -3.92 to 3.68, *P* = 0.95), internal rotation (264 patients; MD = -0.09, 95% CI: -1.49 to 1.31, *P* = 0.90), and forward flexion (118 patients; MD = 1.83, 95% CI: -1.83 to 5.48, *P* = 0.33) all showed non-significant differences. Notably, TOA demonstrated significantly superior improvement in abduction (126 patients; MD = 10.51, 95% CI: 5.29 to 15.72, *P* < 0.0001), with no observed heterogeneity (I² = 0%) (Fig. 4). However, the analysis included a limited number of patients, and whether this magnitude of improvement correlates with meaningful functional gains in daily activities remains to be determined.

### Postoperative pain

**Fig. 5:**
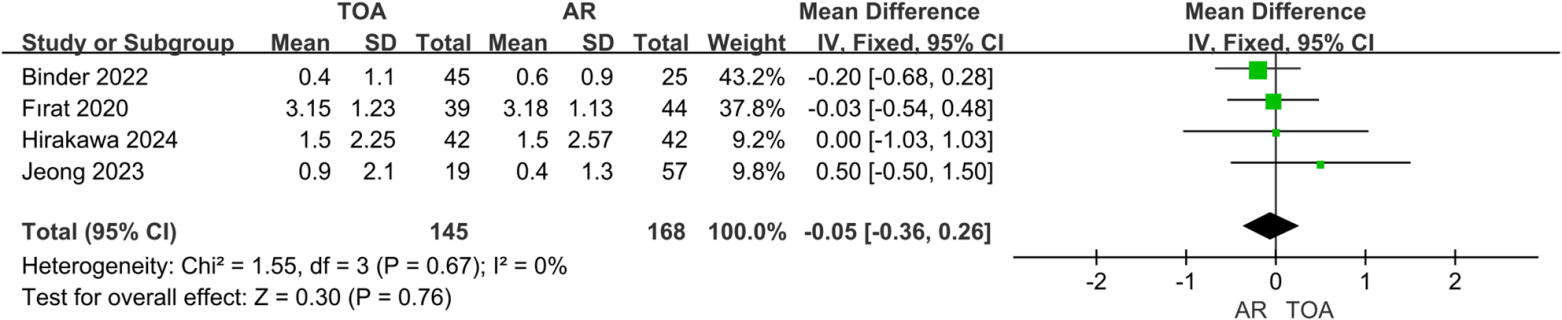
Forest plot comparing VAS pain score between TOA and AR groups

VAS pain score (313 patients) showed no significant difference between TOA and AR groups (MD = -0.05, 95% CI: -0.36 to 0.26, *P* = 0.76), with negligible heterogeneity (I² = 0%) (Fig. 5).

### Structural integrity

**Fig. 6:**
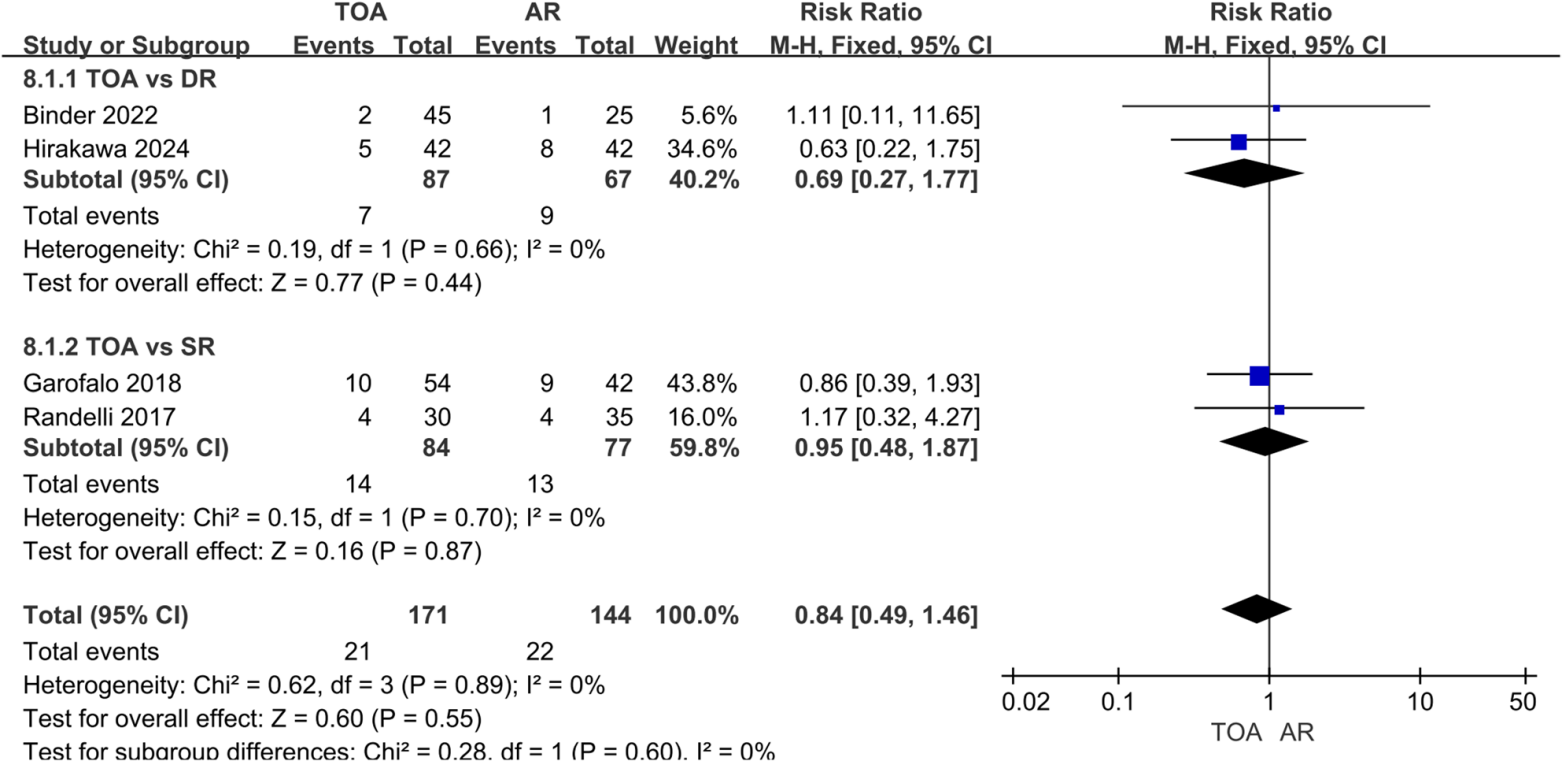
Forest plot comparing retear rate between TOA and AR groups

Assessment of rotator cuff integrity using the Sugaya classification (IV/V indicating retear) revealed comparable retear rates. The overall retear rate was 13.2% in the TOA group versus 15.6% in the AR group, with no significant difference (RR = 0.84, 95% CI: 0.49–1.46, *P* = 0.55; I² = 0%). Subgroup analyses confirmed that no statistically significant difference was observed both when TOA was compared to DR repair (RR = 0.69, 95% CI: 0.27–1.77, *P* = 0.44) and to SR repair (RR = 0.95, 95% CI: 0.48–1.87, *P* = 0.87) (Fig. 6).

## Discussion

This review suggests comparable clinical outcomes between arthroscopic TOA and AR for rotator cuff tears. Pooled data from eight studies (582 patients) revealed no statistically significant differences in functional scores (Constant, ASES), pain assessment (VAS), or overall retear rates between the two techniques. Notably, patients in the TOA group demonstrated significantly superior shoulder abduction function compared to the AR group.

Theoretically, arthroscopic TOA technique, which directly fixes tendons through bone tunnels, provides a larger tendon-bone contact area and more uniform pressure distribution, thereby reducing localized stress concentration caused by anchors. These attributes should theoretically enhance healing and lower retear risk [10, 11]. However, the present analysis did not identify a significant difference in retear rates between groups. Retear risk is strongly associated with factors like advanced age, osteoporosis, larger tear size, and muscle degeneration [26, 27], which may overshadow differences attributable to surgical technique alone.

Subgroup analysis revealed comparable functional and structural outcomes between TOA and DR anchor repair techniques. This similarity may be attributable to a shared biomechanical principle underlying both techniques: the achievement of broad, pressurized contact at the tendon-bone interface [21]. The structural convergence in repair constructs likely underlies their comparable clinical outcomes.

Regarding postoperative pain, while pooled VAS scores showed no long-term difference (*P* = 0.76), the transient advantage of TOA noted by Randelli et al. during weeks 3–4 (*P*<0.05) [24] warrants consideration. This early benefit may be attributed to the avoidance of suture knots-induced subacromial inflammation [28] and the osteoinductive cytokine release from bone tunnels [12]. Crucially, while surgical techniques may differentially impact early pain perception, the comparison of final follow-up VAS scores (typically>12 months) in the included studies likely obscured transient differences, explaining the non-significant pooled results.

Our findings must be interpreted within the context of several limitations. First, the included studies encompassed heterogeneous surgical techniques (e.g., X-box and dual-tunnel for TOA; SR, DR, and suture-bridge for AR), each with distinct biomechanical characteristics. Although subgroup analyses were performed for SR and DR anchor techniques, the variability within TOA methods may obscure nuanced differences in clinical outcomes. This underscores the need for future studies to directly compare standardized technique variants within each repair category. Furthermore, the included studies varied in their reporting and adjustment for important patient-related confounders, such as age, comorbidities (e.g., diabetes), tear size and retraction, tendon quality, muscle fatty degeneration, and repair tension. These factors are known to influence healing and functional recovery after rotator cuff repair.

In summary, this meta-analysis indicates that TOA provides clinical and structural outcomes comparable to those of AR for rotator cuff tears. While TOA showed a significant advantage in postoperative abduction range of motion, no differences were observed in functional scores, pain assessment, or retear rates. Based on the available evidence, TOA can be considered a viable alternative to anchor-based repair techniques.

## Supplementary Information

Supplementary Material 1.

Supplementary Material 2.

## Data Availability

Data from this study are available upon request from the corresponding author.

## Abbreviations

TOA: Transosseous anchorless repair
AR: Suture anchor repair
RCTs: Randomized controlled trials
ROM: Range of motion
VAS: Visual analog scale
MD: Mean differences
RR: Risk ratios
CI: Confidence intervals
ASES: American Shoulder and Elbow Surgeons
SR: Single-row
DR: Double-row
PRISMA: Preferred Reporting Items for Systematic Reviews and Meta-Analyses
MeSH: Medical Subject Headings
RoB 2: Risk of Bias Tool
MRI: Magnetic resonance imaging
