# Supplementary material for: Arthroscopic transosseous anchorless versus suture anchor repair for rotator cuff tears: a meta-analysis
Source: BMC Musculoskelet Disord. 2026 Feb 7;27:206. doi: 10.1186/s12891-026-09557-8 (PMC12977394; doi:10.1186/s12891-026-09557-8)
Supplement: Supplementary file 1 — Supplementary Material 1. [file 12891_2026_9557_MOESM1_ESM.docx]

**Table S1 Take the search in the PubMed database as an example**

| PubMed | | Total |
| --- | --- | --- |
| #1 | "Rotator Cuff"[Mesh] | [9,1](https://pubmed.ncbi.nlm.nih.gov/?sort=date&term=)09 |
| #2 | ((("Rotator cuff"[Title/Abstract]) OR ("Rotator cuff tear"[Title/Abstract])) OR ("Rotator cuff injury"[Title/Abstract])) OR ("Rotator cuff repair"[Title/Abstract]) | [18,077](https://pubmed.ncbi.nlm.nih.gov/?term=((([Title/Abstract])+OR+([Title/Abstract]))+OR+([Title/Abstract]))+OR+([Title/Abstract])&sort=&size=200) |
| #3 | #1 OR #2 | [19,5](https://pubmed.ncbi.nlm.nih.gov/?term=()45 |
| #4 | (((transosseous[Title/Abstract]) OR (anchorless[Title/Abstract]))) OR ("transosseous tunnel"[Title/Abstract]) | [1,804](https://pubmed.ncbi.nlm.nih.gov/?term=(((Transosseous[Title/Abstract])+OR+(anchorless[Title/Abstract])))+OR+([Title/Abstract])&sort=&size=200) |
| #5 | #3 AND #4 | 429 |
